# Genetically Engineered Mouse Models of Pituitary Tumors

**DOI:** 10.3389/fonc.2014.00203

**Published:** 2014-08-01

**Authors:** David A. Cano, Alfonso Soto-Moreno, Alfonso Leal-Cerro

**Affiliations:** ^1^Unidad de Gestión Clínica de Endocrinología y Nutrición, Hospital Universitario Virgen del Rocío, Seville, Spain; ^2^Instituto de Biomedicina de Sevilla (IBiS), Hospital Universitario Virgen del Rocío/Consejo Superior de Investigaciones Científicas/Universidad de Sevilla, Seville, Spain

**Keywords:** pituitary, adenoma, GEMMs, transgenic mice, mouse models

## Abstract

Animal models constitute valuable tools for investigating the pathogenesis of cancer as well as for preclinical testing of novel therapeutics approaches. However, the pathogenic mechanisms of pituitary-tumor formation remain poorly understood, particularly in sporadic adenomas, thus, making it a challenge to model pituitary tumors in mice. Nevertheless, genetically engineered mouse models (GEMMs) of pituitary tumors have provided important insight into pituitary tumor biology. In this paper, we review various GEMMs of pituitary tumors, highlighting their contributions and limitations, and discuss opportunities for research in the field.

## Introduction

Pituitary adenomas occur in 10–15% of the general population as determined by autopsy studies (1, 2). The vast majority are pituitary incidentalomas with no apparent clinical manifestations. However, clinically significant pituitary tumors are associated with significant morbidity and decreased quality of life. Pituitary adenomas may cause symptoms due to abnormal hormone secretion, mass effect, or both. About 50% of pituitary adenomas are prolactinomas that secrete prolactin and cause galactorrhea and hypogonadism. GH-producing somatotropinomas, that cause gigantism and acromegaly, and ACTH-producing corticotropinomas that cause Cushing disease constitute 10 and 5% of pituitary adenomas, respectively. TSH-producing thyrotroph tumors are rare (less that 1%). Clinically non-functioning pituitary adenomas (NFPA) that do not secrete hormones represent about 30% of pituitary adenomas (3, 4).

Pituitary adenomas are usually benign. However, a significant number of pituitary adenomas show an aggressive behavior, with local invasion, increased risk of recurrence after surgery and lack of therapeutic response (5, 6). Malignant pituitary carcinomas are very rare, comprising only 0.2% of all pituitary tumors (7). Non-endocrine tumors can also be found in the pituitary including craniopharyngiomas and blastomas that arise from embryonic remnants of the pituitary (8, 9). Currently available treatment options for pituitary adenomas include drug therapy (mainly dopamine-agonist and somatostatin analogs), surgery, and radiotherapy. However, the currently available therapies fail to control the disease in a significant number of patients emphasizing the need to develop novel therapeutic approaches.

The rarity of pituitary tumors, difficulty to obtain biopsy samples, and lack of human pituitary cell lines pose a significant challenge to pituitary-tumor researchers. Animal models constitute valuable tools for investigating the pathogenesis of pituitary tumors as well as for preclinical testing of novel therapeutics approaches. There are a variety of animal models of pituitary tumors including spontaneous tumors, subcutaneous cell implantation, and genetically engineered mouse models (GEMMs) (transgenic and knockout mice) and each model has its advantages and limitations. In this review, we focus on GEMMs of pituitary tumors (Table 1), highlighting their contributions and limitations, and discuss opportunities for research in the field.

**Table 1:** **Summary of GEMMs for pituitary tumors**.

Model type	Genetic modification	Type of tumor	Tumor latency (months)	Reference
**TRANSGENIC**				
PyLT	Polyoma large T antigen overexpression	Corticotropinoma	9	Helseth et al. (10)
POMC-SV40 large T antigen	SV40 large T antigen overexpression (POMC promoter)	Intermediate lobe	2–3	Low et al. (11)
AVP-SV40 large T antigen	SV40 large T antigen overexpression (AVP promoter)	GH-producing adenomas	–	Stefaneanu et al. (12)
FSHβ-SV40 large T antigen	SV40 large T antigen overexpression (FSHβ promoter)	Null cell adenomas	9	Kumar et al. (13)
hGRHH	GHRH overexpression	Somatotrophs and mammosomatotrophs	10–24	Asa et al. (14)
PRL-TGFα	TGFα overexpression	Prolactinomas	12	McAndrew et al. (15)
PRL-ptd-FGFR4	Overexpression of N-terminally truncated isoform of FGFR4	Prolactinomas	11	Ezzat et al. (16)
αGSU-PTTG1	PTTG1 overexpression	Gonadotroph hyperplasia		Donangelo et al. (17)
CMV-HMGA1	HMGA1 overexpression	Mixed growth hormone/prolactin adenomas	16 (female) 22 (male)	Fedele et al. (18)
CMV-HMGA2	HMGA2 overexpression	Somatotrophs, lactotrophs and mammosomatotrophs	6 (female) 18 (male)	Fedele et al. (19)
**CONVENTIONAL KNOCK OUT**				
*Men1* heterozygous	*Men1* inactivation	Prolactinomas	16	Crabtree et al. (20)
*Men1* heterozygous	*Men1* inactivation	Prolactinomas, NFPA	>9	Loffler et al. (21)
*Men1* heterozygous	*Men1* inactivation	Prolactinomas, GH-producing adenomas	>13	Bertolino et al. (22)
*Men1* heterozygous	*Men1* inactivation	Mammosomatotrophs (90%). ACTH-producing intermediate lobe (10%)	12	Harding et al. (23)
*AIP* heterozygous	*AIP* inactivation	GH-producing adenomas (90%), prolactinomas	6	Raitila et al. (24)
*Drd2* homozygous	*Drd2* inactivation	Prolactinomas	>12 (only females)	Kelly et al. (25), Asa et al. (26)
*Prl* homozygous	*Prl* inactivation	Prolactinomas	8	Cruz-Soto et al. (27)
*Prlr* homozygous	*Prl receptor* inactivation	Prolactinomas	14	Schuff et al. (28)
*Rb* heterozygous	*Rb* inactivation	ACTH-producing intermediate lobe	12	Jacks et al. (29)
*p27^Kip1^* homozygous	*p27^Kip1^* inactivation	Intermediate lobe	12	Kiyokawa et al. (30); Nakayama et al. (31)
*p18^Ink4c^* homozygous	p18^Ink4c^ inactivation	Intermediate and anterior lobe	12	Franklin et al. (32)
Compound *p18^Ink4c^* *p27^Kip1^*homozygous	Double p18^Ink4c^ and *p27^Kip1^* inactivation	Intermediate and anterior lobe	3	Franklin et al. (32)
Cdk4^R24C/R24C^ mutant mice	Mutation rendering the Cdk4 protein insensitive to INK4 inhibitors	Anterior lobe	15	Sotillo et al. (33)
Compound Cdk4^R24C/R24C^ *p27^Kip1^* homozygous mutant mice	Mutation rendering the Cdk4 protein insensitive to INK4 inhibitors and and *p27^Kip1^* inactivation	Poorly differentiated adenomas	2	Sotillo et al. (34)
**CONDITIONAL KNOCK OUT**				
*rGhrhr*-Cre PRKAR1A	Pituitary-specific (somatotrophs, lactotrophs, and thyrotrophs) PRKAR1A inactivation	Somatotrophs, lactotrophs, and thyrotrophs	18	Yin et al. (35)
*POMC-*specific *Rb* heterozygous	*Rb* inactivation in POMC-expressing cells	Intermediate lobe	9	Vooijs et al. (36)
*POMC-*specific *Rb* homozygous	*Rb* inactivation in POMC-expressing cells	Intermediate lobe	3	Vooijs et al. (36)
*POMC-*specific *Rb* homozygous	*Rb* inactivation in POMC-expressing cells	Not reported	6	Vooijs et al. (37)
*Ins-Cre Men1*	*Men1* homozygous inactivation in pituitary due to ectopic Cre expression	Prolactinomas	9	Biondi et al. (38)
*GFAP-Cre Bmi1*	*Bmi1* homozygous pituitary-specific inactivation	Intermediate lobe	>12	Westerman et al. (39)
*Hesx1-Cre;Ctnnb1^lox(ex3)^*^+^ *Sox2-CreERT2;Ctnnb1^lox(ex3)^*^+^	Pituitary-specific overexpression of constitutively active form of β-catenin	Adamantinomatous, craniopharyngioma	3–6	Gaston-Massuet et al. (40) Andoniadou et al. (41)

## Molecular Pathogenesis of Pituitary Tumors

Before we discuss the various GEMMs for pituitary tumors, we will briefly describe the molecular abnormalities in pituitary tumors. For a more comprehensive view of this topic, the reader is directed to several excellent reviews (4, 42–44).

The majority of pituitary adenomas are sporadic. However, approximately 5% of cases arise in the context of familial syndromes (45) and several of the genes involved in these hereditary adenomas have been identified. Multiple endocrine neoplasia type 1 (MEN1) is an autosomal dominant familial disorder with mutations in the tumor suppressor gene MEN1. The cyclin-dependent kinase inhibitor 1B (CDKN1B) is associated with a MEN1-like phenotype now called MEN4. The McCune-Albright syndrome is a rare disease caused by mutations in the GNAS oncogene that encodes the guanine nucleotide-binding protein alpha stimulating activity polypeptide. Inactivating mutations in the PRKAR1A gene that encodes the protein kinase A regulatory subunit-1-alpha are associated with the neoplasia syndrome Carney complex. Familial isolated pituitary adenoma (FIPA) is a rare syndrome defined as familial presentation of pituitary adenomas in the absence of evidence for MEN1 and Carney complex (45). Around 15–40% of FIPA patients harbor germline mutations in the aryl hydrocarbon receptor interacting protein gene (AIP) gene. More recently, it has been suggested that mutations in succinate dehydrogenase genes can predispose to pituitary tumors although the evidence is limited to a single kindred of patients (46).

Pituitary adenomas are thought to be of monoclonal origin according to early studies (47, 48). Despite extensive research on pituitary tumors in the last decade, the molecular mechanisms underlying tumorigenesis in sporadic pituitary tumors remain unclear. There is little evidence to date that the mutations associated to familial pituitary adenomas are involved in most sporadic pituitary adenomas. The *GNAS* gene is the only gene in which mutations have been found in a significant proportion of sporadic pituitary adenomas (30–40% of GH-producing adenomas) although the oncogenic role of such mutations is still unclear (49, 50). Similarly, mutations in classical oncogenes and tumor suppressor such genes TP53 and MYC are only rarely observed, and restricted to aggressive or pituitary carcinomas. However, a significant number of molecular abnormalities, including cell-cycle deregulation, overexpression of growth factors, hormonal overstimulation, epigenetic silenced tumor suppressor genes, and defective signaling pathways have been found in pituitary adenomas although their causative role in pituitary-tumor formation remains to be firmly established (4, 42–44).

Cell-cycle deregulation is considered the main pathogenic event in the formation of pituitary adenomas. It has been estimated that around 80% of human pituitary adenomas display alterations at least in one cell-cycle regulator (51, 52). These alterations include overexpression of cyclins (mainly D1, D3, and E), as well as to downregulation of the cyclin-dependent kinase inhibitor family (mainly p15^Ink4b^, p16^Ink4a^, p18^Ink4c^, p21^Cip1^, and p27^Kip1^) and pRB expression (53). Cyclin D1 and D3 are overexpressed particularly in NFPA while cyclin E is overexpressed mainly in ACTH-producing corticotropinomas. Reduced levels of the INK4 family members of cyclin-dependent kinase inhibitors, p15^Ink4b^, p16^Ink4a^, and p18^Ink4c^ in pituitary adenomas appear to be caused mainly by promoter hypermethylation (54–57). Decreased levels of p27^Kip1^ are common in pituitary carcinomas and ACTH-producing corticotropinomas (58, 59). p21^Cip1^ is also downregulated in NFPAs but, interestingly, appears to be overexpressed in hormone-secreting pituitary adenomas (60). The retinoblastoma tumor suppressor protein (pRb) plays a critical role in G1-S cell-cycle progression. Loss of pRb expression has been found in human sporadic pituitary adenomas (particularly in invasive types) due to loss of heterozygosity or promoter hypermethylation (61, 62). Pituitary-tumor transforming gene 1 (PTTG1) is a member of the securin family and plays an important role in mitosis by controlling sister chromatid separation. PTTG1 is overexpressed in nearly all pituitary adenomas, especially in invasive hormone-secreting pituitary tumors (63). However, its oncogenic role in human pituitary tumorigenesis is unclear. HMGA2 is another oncogene that has been found to be overexpressed in pituitary adenomas (64). More recently, increased expression of HMGA1 in human pituitary adenomas has also been described. This increased expression correlates with tumor invasiveness and proliferation (65). HMGA1 and 2 are members of the high mobility group A (HMGA) protein family, non-histone chromatin binding proteins that act as modulators of cellular processes such as transcription, replication, and recombination. HMGA proteins appear to induce the development of pituitary adenomas through mechanisms involving cell-cycle deregulation.

Several alterations in the expression of growth factors and/or growth factor receptors are frequently observed in pituitary adenomas, including TGFα, EGF, VEGF, and FGF. FGF is one of the best-studied growth factor family in pituitary tumors. FGF-4 is upregulated in pituitary adenomas and its expression correlates with tumor invasiveness. Expression of a N-terminally truncated isoform of FGFR4 (ptd FGFR4) has been found in pituitary adenomas but not in normal pituitary (16). Experimental evidence suggests a tumorigenic role for this isoform in pituitary (16). In contrast, the FGFR2-IIIb isoform appears to be downregulated in pituitary tumors, due to epigenetic modifications (66).

Deregulation of hypothalamic and pituitary feedback mechanisms have long been proposed to participate in pituitary tumorigenesis based on the defects in hormonal signaling observed in pituitary adenomas, mainly in GH- and ACTH-producing adenomas. Thus, increased GHRH expression has been found in aggressive somatotropinomas (67) and pituitary hyperplasia have been observed in patients with endocrine carcinomas ectopically secreting GHRH (68). An inactivating mutation of the GH receptor has also been described in somatotropinomas (69). Differential expression of vasopressin receptors has been reported in ACTH-producing pituitary tumors (70). Also, mutations in the glucocorticoid receptor that reduce glucocorticoid inhibition have been described in corticotroph tumors (71). In prolactinomas, diminished D2 dopamine receptor activity appears to mediate resistance to dopamine-inhibited hormone secretion (72).

Finally, various signaling pathways have been recently implicated in pituitary tumorigenesis. Akt is overexpressed in pituitary adenomas, particularly NFPAs (73) and mutations and amplifications of the phosphoinositide 3-kinase PIK3CA gene have been found in pituitary adenomas (74) suggesting a role for the PI3K/AKT in pituitary adenoma formation. Activation of the Raf/MEK/ERK signaling pathway has also been observed in pituitary adenomas, as demonstrated by the finding that B-Raf is overexpressed, and its downstream effectors MEK1/2 and ERK1/2 overactivated, in pituitary adenomas (75, 76). Increased levels of the hypoxia-inducible factor HIF1α transcription factor have been found in pituitary tumors but not in the normal pituitary (77, 78). Decreased expression of inhibitors of the canonical Wnt pathway has been reported in pituitary adenomas (79). Although these results might suggest the WNT pathway is overactivated in pituitary adenomas, nuclear accumulation of β-catenin was not observed (79). However, activating mutations have been found in the CTNNB1 gene that encodes β-catenin in around 70% of the pediatric form of human craniopharyngioma (adamantinomatous) (80, 81), a pituitary tumor of embryonic origin. Further mechanistic studies are necessary to fully understand the role of these signaling pathways in pituitary-tumor formation.

## Genetically Engineered Mouse models of Pituitary tumors

### Transgenic mice

A common strategy to generate mouse models of cancer is to express viral oncoproteins in the cell/tissue of interest. These animal models based on viral oncogene overexpression provide limited insight into the mechanisms underlying pituitary-tumor formation. However, they can be considered useful tools to study various aspects of pituitary adenomas such as hormone secretion regulation and pathophysiology of hormone excess.

One of the first mouse models of pituitary tumors was generated by expressing the (PyLT) under the control of the polyoma early region promotor. These transgenic mice developed ACTH-producing microadenomas at 9 months of age, large adenomas at 13–16 months of age, and display features of Cushing’s syndrome (10). Several mouse strains generated using oncogenic viruses were generated specifically targeting the pituitary. Thus, expression of the simian virus 40 (SV40) large T antigen under the control of the proopiomelanocortin (POMC) gene promoter induced the formation of pituitary tumors arising from the intermediate lobe. These tumor cells expressed POMC peptides, but not other pituitary hormones (11). SV40 large T antigen overexpression using another promoter, the arginine vasopressin (AVP) gene promoter, also resulted in pituitary tumors although these tumors were composed of undifferentiated somatotroph cells (12). Transgenic mice carrying a temperature-sensitive mutant of simian virus 40 T antigen driven by regulatory elements of the beta subunit of human follicle-stimulating hormone (FSHβ) gene develop pituitary-tumors reminiscent of human null cell adenomas (13).

As mentioned in the previous section, various hypothalamic hormones and growth factors required for normal pituitary function and formation are overexpressed in pituitary adenomas. Transgenic approaches allow the overexpression of these hormones and factors specifically in the pituitary to evaluate their role in pituitary tumorigenesis. Overexpression of the GH-releasing hormone (GHRH) gene in mice results in hyperplasia of pituitary somatotrophs, lactotrophs, and mammosomatotrophs (cells producing both GH and PRL) by 8 months of age that leads to mixed GH and prolactin secreting pituitary adenomas by 10–24 months of age (14). These results indicate that GHRH might promote proliferation of transformed pituitary cells. However, the extended tumor latency period suggests the requirement for additional molecular alterations in the formation of pituitary tumors in these mice. A transgenic mouse model was generated in which TGFα was specifically overexpressed in the pituitary lactotrophs using the prolactin promoter (15). These transgenic mice developed lactotroph hyperplasia at 6 months of age and prolactin-producing pituitary adenomas with high penetrance by 12 months of age. Pituitary lactotroph-specific overexpression of the N-terminally truncated isoform of FGFR4 (ptd-FGFR4), but not the wild-type FGFR4, in mice leads to the development of tumors reminiscent of human prolactinomas (16). Interestingly, ptd-FGFR4 overexpression causes pituitary adenoma formation without preceding hyperplasia. The mechanism by which ptd-FGFR4 might facilitate pituitary cell transformation appears to involve alterations in neural cell-adhesion molecule/N-cadherin signaling that lead to diminished cell-adhesion (82). The oncogenic role of PTTG1 and HMGA2, two oncogenes previously suggested to play a relevant role in human pituitary adenoma formation, has been analyzed in transgenic mice. Targeted PTTG1 overexpression to gonadotroph cells of the pituitary driven by the alpha-subunit glycoprotein (alphaGSU) promoter results in gonadotroph hyperplasia (17). However, only occasional microadenomas were observed. These observations seem to question the tumorigenic potential of PTTG1 in pituitary. However, when overexpressing-PTTG1 mice were crossed with *Rb* heterozygous mice, which develop pituitary tumors with high penetrance (see below), increased frequency of pituitary adenomas was observed. On the contrary, PTTG1 deletion diminishes pituitary-tumor development in *Rb* heterozygous mice (83). Taken together, these results obtained from genetically modified mice indicate that PTTG1 facilitates pituitary adenoma formation. Transgenic mice expressing high levels of the HMGA2 transgene in all tissues (by using the cytomegalovirus promoter) developed mixed growth hormone/prolactin cell pituitary adenomas with high penetrance (85% of female animals) by 6 months of age (19). Of note, transgenic males developed pituitary adenomas with a lower penetrance (40%) and a longer latency period (about 18 months). Interestingly, HMGA1 overexpression also leads to the development of pituitary adenomas secreting prolactin and GH (18). Subsequent studies performed in these transgenic mice have shown that HMGA proteins mediate the formation of these tumors through a mechanism that involves the Rb/E2F1 pathway, thus further supporting a critical role of this pathway in pituitary tumorigenesis (84).

Altogether, transgenic mice overexpressing proteins in the pituitary have provided important insights into pituitary tumorigenesis. One of the major limitations of these mouse models is the regulation of the expression of the transgene that ideally should be limited to adult stages, thus mimicking the human situation.

### Conventional knockout models

The use of conventional or whole-body knockout mouse models provides a different approach for analyzing the function of important genes in pituitary tumorigenesis. Thus, conventional knockout mice are a crucial tool to analyze the role of tumor suppressor genes in pituitary tumorigenesis. Conventional knockout mice have been generated to assess the direct effect of several genes linked to familial pituitary tumors such as MEN1, CDKN1B, and AIP in pituitary-tumor development. Four different *Men1* knockout mouse strains have been generated (20–23). Homozygous *Men1* mice are embryonic lethal due to defects in craniofacial development but heterozygous *Men1* mice are viable and have been extensively analyzed for tumor formation. In general, all these *Men1* heterozygous mouse models correlate closely with the human MEN1 phenotype regarding tumor incidence and spectrum. These mice develop tumors of various endocrine tissues, including pancreatic islet cell tumors, pituitary and parathyroid adenoma, and adrenocortical tumors. Loss of heterozygosity and menin expression was demonstrated in the tumors, consistent with a tumor suppressor role for the *Men1* gene. Furthermore, a higher occurrence of anterior pituitary tumors is observed in female *Men1* heterozygous mice, similar to what it is observed in MEN1 patients. However, the different *Men1* mouse models also show slight phenotypic differences including the type of pituitary adenomas developed. Thus, only prolactin producing adenomas were observed in the *Men1* mouse model generated by Crabtree et al. (20) while prolactinomas and non-secreting adenomas were found in the model generated by Loffler et al. (21). Bertolino et al. (22) reported the formation of prolactinomas and GH-secreting tumors. Finally, Harding et al. (23) reported that the majority the pituitary tumors originated from the pars distalis and contained both prolactin and GH while around 10% of the tumors originated from the pars intermedia and contained ACTH. The reason for these differences in pituitary-tumor formation is unclear but it might be related to the types of *Men1* mutations (different exons were targeted) and/or differences in genetic background.

Recently, a conventional knockout mouse deficient in the AIP, a gene linked to familial isolated pituitary adenoma (FIPA), and isolated familial somatotropinomas have been generated. Homozygous knockout *Aip* mice died during embryogenesis, likely due to cardiovascular abnormalities (24). Heterozygous *Aip* mice were highly prone to pituitary adenomas, developing tumors as early as at 6 months of age (24). Thus, unlike in humans in whom *AIP*-associated tumors often develop in childhood or early adulthood (85), heterozygous *Aip* mice develop adenomas in adulthood. Another major difference between the *Aip* mouse model and the human disease is the complete penetrance of pituitary tumors in the *Aip* mouse model (at 15 months of age). The majority of adenomas found in the *Aip* mouse model were somatotropinomas, although mixed GH/prolactin, prolactinomas, and ACTH-corticotropinomas were also found. Biallelic inactivation of *AIP* was demonstrated in these tumors.

Analysis of conventional knockout mice has also been useful to analyze the effect of disruption of feedback signaling in pituitary-tumor pathogenesis. Thus, analysis of dopamine receptor 2 (*Drd2*) knockout mice indicates that functional inactivation of this receptor might contribute to the formation of pituitary adenomas, namely prolactinomas. Female *Drd2* knockout mice develop massive lactotroph hyperplasia by 12 months of age and invasive prolactinomas at older ages. (25, 26). Male *Drd2* knockout mice develop microadenomas in the absence of hyperplasia. The effects of disrupted prolactin feedback on pituitary tumorigenesis have been demonstrated in knockout mice deficient for prolactin and prolactin receptor (*Prlr*). Prolactin knockout mice develop lactotroph hyperplasia by 6 weeks of age and prolactinomas by 8 months of age (27). Inactivation of *Prlr* also results in lactotroph hyperplasia and formation of invasive adenomas (28).

Studies performed in mice with targeted disruption of cell-cycle regulators collectively support the notion that cell-cycle dysregulation is central in pituitary tumorigenesis (51). Mice heterozygous for *Rb* develop ACTH-producing aggressive tumors arising from the intermediate lobe of the pituitary with high penetrance by 12 months of age (29). Subsequent studies have shown that pituitary-tumor incidence and histological type in *Rb* heterozygous mice is dependent on the mouse strain suggesting an effect of modifier genes in pituitary adenoma formation (86). Homozygous mice deficient for the cyclin-dependent kinase inhibitors p27^Kip1^ and p18^Ink4c^ also develop pituitary adenomas. Homozygous p27^Kip1^mice develop tumors from the intermediate lobe by 12 months of age (30, 31) while p18^Ink4c^ mice develop pituitary adenomas in both the intermediate and anterior lobes (32). Inactivation of other cyclin-dependent kinase inhibitors that are downregulated in human pituitary adenomas, such as p21^Cip1^, p15^Ink4b^, and p16^Ink4a^, does not result in pituitary tumors in mice. The generation of mice with combined inactivation of different cyclin-dependent kinase inhibitors has revealed a cooperation of these cyclin-dependent kinase inhibitors in pituitary-tumor formation. Thus, deletion of p21^Cip1^, p27^Kip1^, or p16^Ink4a^ in combination with loss of p18^Ink4c^ results in increased incidence and decreased latency of pituitary tumors compared to single mutants (32, 87, 88). Of note, double p18^Ink4c^ p27^Kip1^ mutant mice appear to die by 3 months of age due to pituitary adenomas (32). The relevance of INK4 family proteins in pituitary adenoma formation is further supported by the analysis of knock-in mice, which express a CDK4 variant with a mutation that renders the Cdk4 protein insensitive to INK4 inhibitors (Cdk4^R24C/R24C^ mutant mice) (33, 89). These Cdk4^R24C/R24C^ knock-in mice develop multiple tumors including pituitary tumors (25% of penetrance) arising in the anterior pituitary by 15 months of age. A dramatic cooperation in pituitary-tumor formation is observed in double Cdk4^R24C/R24C^ p27^Kip1^ mutant mice, which develop poorly differentiated pituitary tumors that kill these mutant mice in 8–10 weeks of age (34). Cooperation between cyclin-dependent kinase inhibitors and Rb in pituitary-tumor formation has also been observed. Inactivation of p27^Kip1^ and p21^Cip1^in *Rb* heterozygous mice results in decreased latency of pituitary tumors (90, 91). Similarly, loss of p16^Ink4a^ strongly accelerates intermediate lobe pituitary tumorigenesis in *Rb* heterozygous mice (92).

The usefulness of conventional knockout mice as models for evaluating therapeutic agents is highlighted by studies performed with bromocriptine, a dopamine agonist used to treat prolactinomas. Thus, it has been reported that bromocriptine decreases prolactin serum levels in *Prlr* mutant mice harboring tumors (28) and reduces tumor cell proliferation in p27^Kip1^ deficient mice (93). Bromocriptine does not affect prolactin levels in *Drd2* knockout mice, as expected from the absence of the dopamine receptor 2 (28) and thus, these mice can be considered a useful model for dopamine-resistant prolactinomas. The effectiveness of antiangiogenic treatments in dopamine-resistant prolactinomas has been analyzed in *Drd2* knockout mice. Anti-VEGF strategies resulted in prolactin inhibition and decreased tumor cell proliferation in these mice (94).

The mouse models discussed above illustrate how conventional knockout mice help to elucidate the causal role of genes that were predicted to be important for human pituitary tumorigenesis. These conventional knockout mice can also be useful as preclinical models for testing of therapeutic agents.

### Conditional knockout models

The development of conditional gene targeting has allowed to overcome some of the limitations of conventional knockout mice such as potential embryonic lethality or complications due to systemic effects of gene inactivation. The most widely used conditional system is based on the Cre-loxP technology. Combinations of Cre*-loxP* systems, specific promoters, and inducible systems (i.e., tamoxifen) allow tissue- and time-specific inactivation of the gene of interest. The number of pituitary-specific Cre lines available is rapidly increasing (35, 95–102). The generation of pituitary cell-specific Cre transgenic lines has been instrumental in advancing our understanding of pituitary cell function and cell lineages. However, to date, relatively few studies have exploited these Cre lines to evaluate the role of genes of interest in pituitary tumorigenesis.

To explore the role of inactivation of the Carney complex gene PRKAR1A on pituitary tumorigenesis, a pituitary-specific knockout of this gene was generated by crossing mice with conditional alleles of *Prkar1a* with a mouse line expressing the Cre recombinase in pituitary cells using the GHRH receptor promoter (*rGhrhr*-Cre line) (35). This promoter, drives Cre-mediated recombination in pituitary cells of the *Pit1* lineage, including somatotrophs, lactotrophs, and thyrotrophs. Pituitary-specific *Prkar1a* knockout mice exhibited a moderate increased frequency of pituitary adenomas, although not until advanced age (18 months). These tumors were composed of cells that expressed GH, prolactin, and TSH. Using a different conditional system based on the Flp recombinase, specific inactivation of *Rb* in pro-opiomelanocortin-expressing cells has been achieved. Mice in which only an allele of Rb was conditionally inactivated in the pituitary displayed atypical lesions in the intermediate lobe of the pituitary by 6–12 weeks of age that resulted in adenoma formation as the mice aged. Accelerated adenoma formation was observed in mice homozygous for the conditional alleles of *Rb*. These mice developed pituitary adenomas with 100% incidence as early as 12 weeks of age and succumbed to these adenomas by 20 weeks of age (36). The adenomas originated from the intermediate lobe melanotrophs and were indistinguishable from the adenomas observed in conventional *Rb* heterozygous mice. Pituitary-specific inactivation of Rb has also been achieved using a mouse strain in which Cre expression is under the control of the POMC gene promoter. These mice developed pituitary adenomas with 100% incidence after a relatively short median latency (around 6 months) (37). However, no histological characterization of the adenomas was reported. Interestingly, in this study pituitary adenoma formation *in vivo* was monitored by crossing conditional *Rb* knockout mice with transgenic mice that express luciferase under the control of the POMC promoter detecting tumor growth as early as 8 weeks of age.

Pituitary adenomas have been also reported in conditional mouse models using non-pituitary-specific Cre lines. Thus, conditional inactivation of the MEN1 gene using a mouse line in which Cre expression was driven by the insulin promoter resulted in the formation of pituitary adenomas (in addition to islet tumors) by 12 months of age (38). All pituitary adenomas were derived from the pars distalis, as determined by expression of prolactin. The formation of pituitary adenomas in these mice was attributed to aberrant Cre expression in the pituitary. Conditional activation of the polycomb ring finger oncogene using a GFAP-Cre that drives expression in neural and glial lineages developed intermediate and anterior lobe pituitary tumors positive for ACTH and beta-endorphin although with incomplete penetrance and long latency period (1 year) (39). Consistent with these results, Cre expression in the GFAP-Cre line was observed in the pituitary. However, it is unclear whether this Cre activity in the pituitary represents endogenous GFAP expression or ectopic expression of the Cre transgene.

The usefulness of conditional knockout mouse models in the study of pituitary-tumor formation is perhaps best illustrated by two recent studies that analyzed the role of Wnt/β-catenin signaling pathway in pituitary tumorigenesis. Expression of a degradation-resistant mutant form of β-catenin in embryonic pituitary using the *Hesx1-Cre* line was shown to cause the formation of tumors resembling human adamantinomatous craniopharyngioma (ACP) (40). ACP is a type of pediatric non-hormone-producing tumor derived from pituitary embryonic tissue associated with significant morbidity and mortality (8). Interestingly, expression of mutant β-catenin in differentiated cells in the embryonic or adult pituitary using specific Cre lines (Gh-Cre, Prl-Cre, and Pit1-Cre) did not give rise to tumors (40). Further studies from the same group using specific a tamoxifen-inducible Cre line demonstrated that activation of mutant β-catenin in *Sox2*-expressing adult pituitary stem cells also result in the formation of ACP-like tumors (41). The molecular characterization of the tumors formed in these mouse models of ACP have uncovered a number of genes and pathways potentially involved in the formation of ACP including the SHH, BMP, and FGF pathways (103). These findings have been validated in human ACP (103) in what is an excellent example of how genetic studies in mice harboring conditional mutations, in addition to providing mechanistic insight into pituitary tumorigenesis, can lead to the generation of new animal models for modeling human disease.

## Conclusion and Future Directions

Although current GEMMs of pituitary tumors exhibit, to some degree, biochemical and molecular features of pituitary adenomas, no current mouse model exists that fully recapitulates pituitary-tumor formation. The ideal mouse model of cancer faithfully recapitulates the genetic lesions found in human disease. However, the primary genetic defect/s for pituitary tumorigenesis remains unclear. Furthermore, the pathogenic mechanisms for tumor formation may be specific for the different pituitary adenoma subtypes thus, making it a challenge to model pituitary tumors in mice. Despite these limitations, current GEMMs of pituitary tumors have provided important insight into pituitary-tumor biology. Transgenic and knockout mice have proven to be a powerful tool to examine the role of genes predicted to be important for human pituitary tumorigenesis. Thus, the use of knockout mice has validated the putative oncogenic role of genes linked to familial pituitary adenomas such as MEN1 and AIP. Also, the analysis of knockout mice deficient in cell-cycle regulators such as Rb and cyclin-dependent kinase inhibitors have provided evidence that cell-cycle deregulation may be a critical event in pituitary tumorigenesis. As the number of genes found to be deregulated in pituitary adenomas continues to increase, we can expect to see the generation of new mouse models with deletion or overexpression of those genes in the near future. The recent generation of pituitary-specific Cre-expressing mouse strains will certainly have a significant impact in this area since it will permit the analysis of genes in a cell type-specific and temporal fashion thus circumventing problems often associated to conventional knock out mice such as embryonic lethality. Nevertheless, a more detailed characterization of current GEMMs for pituitary tumors may still provide additional valuable insight into pituitary tumorigenesis. For example, in the vast majority of GEMMs of pituitary tumor, adenomas arise after a long latency period suggesting the requirement of additional genetic events. The identification of such events might uncover underlying mechanisms of adenoma formation. Further studies in existing GEMMs may also help to address important specific questions on pituitary tumors that cannot be easily addressed in human such as the cell of origin for pituitary adenomas, interaction between tumor cells and tumor microenvironment, and mechanisms underlying adenoma invasiveness.

Ultimately, the goal of generating faithful GEMMs of cancer is to evaluate therapeutic strategies. However, there is a scarcity of studies evaluating current available therapies for pituitary tumors in GEMMs. Such studies are fundamental to determine the utility of GEMMs of pituitary tumors for preclinical testing of new therapeutic drugs. In this regard, the use of non-invasive imaging to assess monitoring of tumor growth and response to therapy can enhance the utility of these mouse models for preclinical testing as previously shown in Rb conditional knock out mice (37).

As our understanding of the molecular mechanisms involved in pituitary adenoma formation improves, we can expect the generation of more sophisticated and diverse GEMMs that will undoubtedly facilitate research in pituitary tumorigenesis, the identification of biomarkers of tumor aggressiveness and preclinical testing of novel therapies.

## Conflict of Interest Statement

The authors declare that the research was conducted in the absence of any commercial or financial relationships that could be construed as a potential conflict of interest. The Guest Associate Editor Amancio Carnero declares that, despite being affiliated to the same institution as authors David A. Cano, Alfonso Soto-Moreno, and Alfonso Leal-Cerro, the review process was handled objectively and no conflict of interest exists.
